# Blood-Based Biomarkers as Prognostic Factors of Recurrent Disease after Radical Cystectomy: A Systematic Review and Meta-Analysis

**DOI:** 10.3390/ijms24065846

**Published:** 2023-03-19

**Authors:** Heidemarie Ofner, Ekaterina Laukhtina, Melanie R. Hassler, Shahrokh F. Shariat

**Affiliations:** 1 Department of Urology, Comprehensive Cancer Center, Medical University of Vienna, 1090 Vienna, Austria; heidemarie.ofner@meduniwien.ac.at (H.O.);; 2 Institute for Urology and Reproductive Health, Sechenov University, 119991 Moscow, Russia; 3 Hourani Center for Applied Scientific Research, Al-Ahliyya Amman University, Amman 19328, Jordan; 4 Department of Urology, University of Texas Southwestern Medical Center, Dallas, TX 75390, USA; 5 Department of Urology, Second Faculty of Medicine, Charles University, 150 06 Prague, Czech Republic; 6 Department of Urology, Weill Cornell Medical College, New York, NY 10065, USA; 7 Karl Landsteiner Institute of Urology and Andrology, 1090 Vienna, Austria

**Keywords:** urothelial carcinoma, radical cystectomy, prognostic factor, recurrence, neutrophil-to-lymphocyte ratio, biomarker

## Abstract

Survival outcomes after radical cystectomy (RC) for bladder cancer (BCa) have not improved in recent decades; nevertheless, RC remains the standard treatment for patients with localized muscle-invasive BCa. Identification of the patients most likely to benefit from RC only versus a combination with systemic therapy versus systemic therapy first/only and bladder-sparing is needed. This systematic review and meta-analysis pools the data from published studies on blood-based biomarkers to help prognosticate disease recurrence after RC. A literature search on PubMed and Scopus was conducted according to the Preferred Reporting Items for Systematic Reviews and Meta-analyses (PRISMA) statement. Articles published before November 2022 were screened for eligibility. A meta-analysis was performed on studies investigating the association of the neutrophil-to-lymphocyte ratio (NLR), the only biomarker with sufficient data, with recurrence-free survival. The systematic review identified 33 studies, and 7 articles were included in the meta-analysis. Our results demonstrated a statistically significant correlation between elevated NLR and an increased risk of disease recurrence (HR 1.26; 95% CI 1.09, 1.45; *p* = 0.002) after RC. The systematic review identified various other inflammatory biomarkers, such as interleukin-6 or the albumin-to-globulin ratio, which have been reported to have a prognostic impact on recurrence after RC. Besides that, the nutritional status, factors of angiogenesis and circulating tumor cells, and DNA seem to be promising tools for the prognostication of recurrence after RC. Due to the high heterogeneity between the studies and the different cut-off values of biomarkers, prospective and validation trials with larger sample sizes and standardized cut-off values should be conducted to strengthen the approach in using biomarkers as a tool for risk stratification in clinical decision-making for patients with localized muscle-invasive BCa.

## 1. Introduction

Radical cystectomy (RC) affords a sustained local and distant disease control for patients with muscle-invasive bladder cancer (BCa) [1,2,3]. Nevertheless, overall recurrence-free survival (RFS) at 5 and 10 years after RC are only 68% and 66%, respectively [4]. Prognosis after RC is dependent on histopathologic features such as tumor stage and grade, metastasis status, lymph node involvement, histopathologic subtype, or lymphovascular and blood vessel invasion [5,6,7,8,9]. Given the morbidity and mortality of RC as well as its high recurrence rates, a better preoperative risk stratification and prognostic tool to select the optimal treatment strategy (i.e., local only versus local plus systemic therapy) are needed for optimal clinical decisions [3,10,11].

Much research has arisen on blood-based biomarkers to help guide clinical decision making in muscle-invasive BCa [12,13,14]. Despite substantial efforts, no biomarker is standard in the clinical setting so far, as the majority of studies suffer from inadequate study designs, analysis and reporting [15]. To assess the clinical role of biomarkers, predictive models are needed to show a significant improvement of performance, and the studies reporting a new biomarker should be highly regulated and well planned. As reported by Shariat et al., a study investigating biomarkers should be designed in such a way that it comprises and considers preclinical testing, assay development, feasibility and clinical prevalence, validation and standardization for clinical utility, independent confirmation studies, and impact assessments [15]. This standardized approach is often missing in the protocols of published studies in the past [16].

Since cytokine imbalances and inflammatory responses are described to act as a trigger for urothelial cancer cell proliferation and metastasis formation, blood levels of inflammatory cells seem easily accessible biomarkers that have become increasingly attractive in recent years [17,18,19]. Among these, the neutrophil-to-lymphocyte ratio (NLR) gained special interest as a marker of systemic inflammation, and several articles have been published reporting the NLR as a prognostic tool for survival outcomes in BCa patients [20,21,22].

The aim of this systematic review and meta-analysis is to pool the existing literature assessing the role of blood-based biomarkers regarding disease recurrence in BCa patients after RC because these are available preoperatively and may, therefore, help guide decision making.

## 2. Methods

### 2.1. Search Strategy

To define the research question, we used the PICO format and searched for all studies that included BCa patients (P) who underwent RC (I) and studies that reported prognostic factors (O), comparing patients with disease recurrence to those who did not have recurrence (C) [23]. The keywords used in our search strategy therefore included (((bladder cancer) AND ((cystectomy) OR (radical operation))) AND (prognostic)) AND (recurrence).

The electronic databases PubMed and Scopus were searched according to the PRISMA statement for articles published before November 2022. The study was registered on PROSPERO (ID CRD42023387840) [24].

### 2.2. Study Inclusion and Exclusion

**Figure 1:**
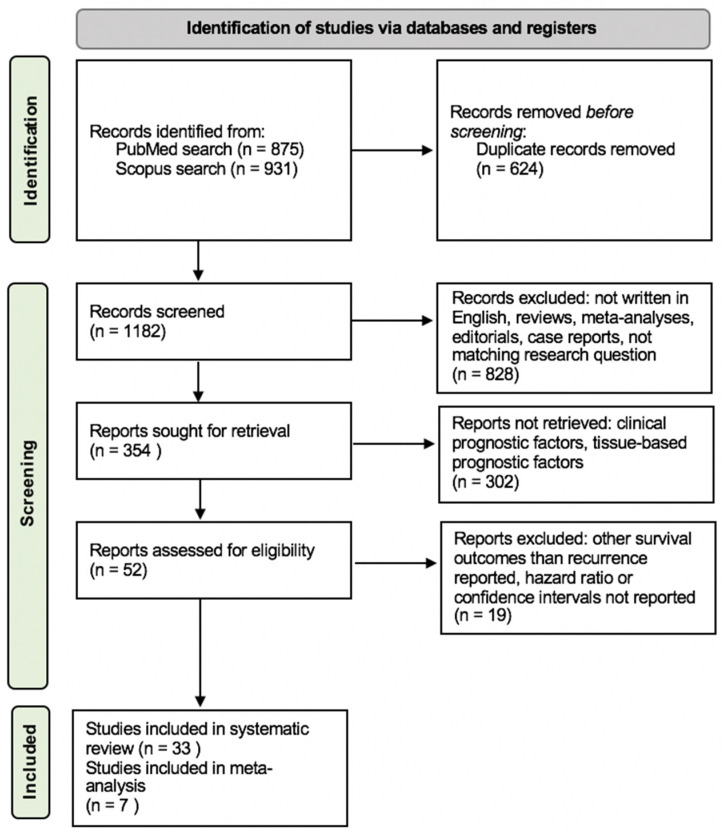
Preferred Reporting Items for Systematic Reviews and Meta-Analyses (PRISMA) 2020 flow diagram. From: Page et al. [25].

Two independent reviewers screened the detected studies initially by titles, then by abstracts. Afterwards, a full-text review was performed. Articles not written in English, case reports, editorials, reviews, and meta-analyses were excluded during the initial screening process, as demonstrated by Figure 1. During the conducted full-text review, publications not matching our research question were additionally excluded and the remaining articles were screened for content concerning blood-based prognostic factors. Articles with clinical factors or tissue-based biomarkers as prognostic tools were not retrieved. For the meta-analysis, the remaining articles were filtered for describing preoperatively measured NLR as a prognostic factor of recurrence. Articles not reporting recurrence or RFS as an endpoint or not stating hazard ratios (HRs) and confidence intervals (CIs) were excluded. References of selected studies were also screened for potentially relevant articles.

### 2.3. Data Extraction

One reviewer conducted the data extraction independently into a datasheet and the process was verified by a second reviewer. Discrepancies were resolved by consensus between co-authors. Data containing the following information were obtained: name of the first author; year of publication; whether a univariable and/or multivariable analysis was conducted; sample size, gender and the median age of subjects; median follow-up; HR in multivariable analysis with a CI of 95%; *p*-values; cut-off values and the timepoint of measurement; and whether neoadjuvant chemotherapy or radiation was received. The primary endpoint investigated was recurrence or RFS; other reported survival outcomes were also listed in the data sheet.

### 2.4. Risk of Bias Assessment

We evaluated the risk of bias using the risk of bias in non-randomized studies of interventions (ROBINS-I) tool, which is based on seven domains: bias due to confounding, participant selection, classification of interventions, deviations from intended intervention, missing data, measurement of outcomes, and selection of the reported results [26]. The detailed data are added as Supplementary Table S1.

### 2.5. Statistical Analysis

To perform statistical analysis, data were extracted from the collected articles eligible for the meta-analysis on NLR only (n = 7), as described above. In addition, *p*-values < 0.05 were considered statistically significant. Heterogeneity among the outcomes of included studies in this meta-analysis was evaluated using Cochrane’s Q test and the I^2^ statistic. Significant heterogeneity was indicated by a *p* < 0.05 in Cochrane’s Q tests and a ratio > 50% in I^2^ statistics. A random effect model was used in case of heterogeneity. The statistical analysis was performed with Review Manager version 5.4.1 (The Cochrane Collaboration, London, UK).

## 3. Results

### 3.1. Search Results

In total, 1806 studies published between 1976 and 2022 were found during the literature search. Duplicates were removed and 1182 publications were screened by title and abstract, leaving 354 articles. As shown in Figure 1, thirty-three articles were eligible for the systematic review and seven articles for meta-analysis.

### 3.2. Study Characteristics

Thirty-three articles reporting pre-treatment blood-based biomarkers as prognostic factors for disease recurrence in BCa patients treated with RC were retrieved for this systematic review. The characteristics of the included studies are listed in Table 1. The studies were published between 2004 and 2022, and the sample size ranged between 26 and 4335 patients. Six research groups conducted prospective single-center studies, whereas the other articles reported retrospective data analyses. Thirteen of the eligible studies investigated inflammatory biomarkers as a prognostic factor of survival outcomes. All eligible studies reported RFS as an outcome, whereas cancer-specific survival (CSS) and overall survival (OS) were reported by 23 and 28 articles, respectively.

### 3.3. Systematic Review of Blood-Based Biomarkers

#### 3.3.1. Inflammatory Biomarkers

Thirteen eligible articles investigated inflammatory biomarkers in patients treated with RC for BCa. Schuettfort et al. showed an association of elevated interleukin-6 and its soluble receptor with RFS, with a HR of 1.04 (95% CI 1.03–1.05) and 1.32 (95% CI 1.23–1.41), respectively. Their attempt to create a prognostic panel of systemic inflammatory response (SIR) biomarkers as a tool for patient selection failed, however, as it did not improve prognostic accuracy beyond the established clinicopathologic characteristics [47,53]. Besides the already mentioned inflammatory markers, the lymphocyte-to-monocyte ratio and lymphocyte-to-platelet ratio have been investigated as prognostic factors in different publications [20,39]. Grossmann et al. calculated a systemic inflammatory index (SII) by using neutrophil, platelet, and lymphocyte counts [48]. A high preoperatively measured SII was associated with worse RFS, OS, and CSS, but decision curve analysis did not show a clinical net benefit for decision-making.

Another inflammatory marker investigated by Fallah et al. is the presence of myeloid-derived suppressor cells, which are reported to increase through inflammatory cytokines released by cancer cells [40]. An elevated monocytic myeloid-derived suppressor cell count in blood was shown to be a negative prognostic factor for OS and RFS (HR 5.95, *p* = 0.0004 and HR 7.487, *p* = 00004, respectively). A low albumin-to-globulin ratio as a sign of systemic inflammation was reported to be negatively associated with RFS, OS, and CSS by two studies [35,43].

Therefore, abnormal levels of inflammatory biomarkers seem to have a negative correlation with oncologic outcomes after RC for BCa, but the attempts of creating prognostic panels for clinical decision-making failed due to the lack of a clinical added value of these biomarkers.

#### 3.3.2. Serum Carbohydrate Antigen and Carcinoembryonic Antigen

Serum carbohydrate antigen (CA) has been studied in several articles [27,28,31]. Kouba et al. report that higher mean CA-125 serum levels in patients with pT2/T3 BCa were associated with recurrence after RC (20.1 vs. 10.8 U/mL, respectively; *p* = 0.224) [28]. Contradictory to these results, Chang et al. and Ahmadi et al. did not find an association between the preoperative blood levels of CA-125 or carcinoembryonic antigen (CEA) with RFS after RC [27,31]. Ahmadi et al. reported a negative association between high preoperative CA 19-9 levels and 3-year RFS in patients with BCa.

#### 3.3.3. Circulating Tumor Cells and DNA

Christensen et al. investigated the prognostic impact of circulating tumor DNA in BCa patients and reported an overall recurrence rate after RC of 76% and a 12-month recurrence rate of 59% in ctDNA-positive patients [37]. The recurrence rate for ctDNA-negative patients was 0% for both time points. The published results seem very promising, yet the study was conducted with a small sample size (n = 68) and needs further validation.

The expression of the human epidermal growth factor receptor 2 (HER-2) on circulating tumor cells was assessed by Rink et al. through fluorescein-labeled antibodies [30]. In 23% of patients, circulating tumor cells were detected and 25% of subjects with HER-2 positive tumors expressed HER-2 on their circulating tumor cells. The presence of circulating tumor cells was associated with a worse RFS, CSS, and overall mortality (OM), but a correlation between HER-2 expression and clinical outcomes was not reported and the study was limited by a small sample size (n = 100).

#### 3.3.4. Nutritional Status Biomarkers

Three studies reported the association of impaired nutritional status with worse survival outcomes [34,39,44]. Higher albumin-to-alkaline phosphatase ratios, as a sign of better nutritional status, correlated with better RFS, OS, and CSS. Djaladat et al. reported that higher serum albumin levels and the American Society of Anesthesiologists (ASA) score were both associated with a higher 90-day complication rate after radical surgery. Similarly, Ninomiya et al. demonstrated that a worse prognostic nutritional status was associated with a higher recurrence rate (*p* = 0.028) [39].

#### 3.3.5. Factors of Angiogenesis and Vascular Endothelial Growth

Three articles investigating different markers associated with angiogenesis and vascular endothelial growth were identified. Laukhtina et al. conducted a retrospective analysis of 1036 patients, showing that a higher preoperatively measured plasma level of endoglin was associated with worse RFS (HR 1.85, *p* < 0.001), CSS (HR 2.02, *p* < 0.001), and OS (HR 1.63, *p* < 0.001) [49].

Similarly, higher preoperative plasma levels of both vascular cell adhesion molecule-1 (VCAM-1) and the vascular endothelial growth factor (VEGF) were associated with worse RFS, CSS, and OS after RC [42,50].

#### 3.3.6. Other Blood-Based Biomarkers

The level of urokinase plasminogen activator proteins correlates with aggressive disease and worse survival outcomes after RC for BCa, as reported by Dohn et al. and Schuettfort et al. [42,46]. Hazza et al. measured serum and urine clusterin levels and showed a negative correlation with recurrence and OS [29].

Sari Motlagh et al. investigated the prognostic impact of the insulin-like growth factor-I and its binding proteins 2 and 3 in another retrospective cohort study [50]. There was no association between the insulin-like growth factor-I and clinical outcomes, but the authors report a favorable prognosis in subjects with elevated binding proteins. Katayama et al. and Yuk et al. proposed the integration of the hepatocyte growth factor and the De Ritis ratio into a prognostic panel for risk stratification [38,55].

Another interesting blood-based biomarker is serum lactate dehydrogenase. Since cancer cells prioritize glycolysis and lactate dehydrogenase catalyzes pyruvate and lactic acid, the enzyme’s serum level might reflect the metabolic changes seen in tumor progression. An elevated lactate dehydrogenase level was associated with a shorter disease-free survival (HR 2.051; *p* = 0.019) [41]. With its feasibility and easy access, the measurement of serum lactate dehydrogenase might therefore be a promising part of a risk stratification panel.

### 3.4. Meta-Analysis

The seven studies eligible for meta-analysis were published between 2014 and 2021, as presented in Table 2. All of them report preoperatively measured NLR as a prognostic factor of RFS. The sample sizes ranged between 84 and 4198 patients, reporting 6388 patients in total. The median follow-up time ranged from 30.1 months to 10.9 years. Two studies did not report their median follow-up times. The patient median age ranged between 67 and 79 years. More male than female subjects were included in the eligible studies. Except for one, all studies reported statistically significant results regarding disease recurrence. The reported NLR cut-off values ranged between 2.6 and 3. Study characteristics in more detail are listed in Supplementary Table S2.

**Figure 2:**
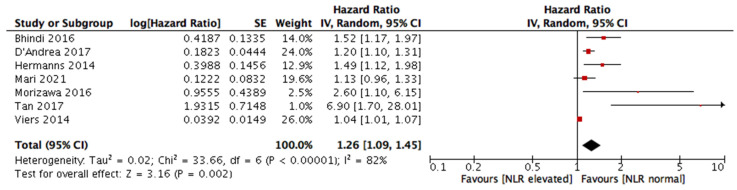
Meta-analysis results of neutrophil-to-lymphocyte ratio (NLR) and its impact on the recurrence-free survival (RFS) of urothelial carcinoma patients after RC [20,21,22,32,33,36,45].

The meta-analysis reports worse RFS for patients with an elevated preoperative NLR with a HR of 1.26 (95% CI 1.09, 1.45), *p* = 0.002. Since a statistically significant heterogeneity (I^2^ = 82%) was shown between studies, a random effect model was used. The statistical results visualized by a forest plot are shown in Figure 2.

## 4. Discussion

This systematic review summarizes the available data of the last two decades on preoperative blood-based biomarkers as prognostic factors of recurrence in RC patients, with special focus on inflammatory biomarkers. In our meta-analysis, we confirmed the strong prognostic impact of preoperative NLR on RFS after RC.

We identified various blood-based biomarkers as predictors of recurrence after RC for BCa through a systematic review. Inflammatory biomarkers were reported to be negatively correlated with recurrence, but attempts to create prognostic panels for clinical decision-making have failed [48,53]. Two studies evaluated the presence of ctDNA and circulating tumor cells with promising results, but were limited by a small sample size [30,37]. Further reports evaluated serum carbohydrate antigen, nutritional status, and factors of angiogenesis as prognostic biomarkers.

Inflammation and cancer have been linked since Rudolf Virchow reported the presence of leukocytes in tumor biopsies in 1863 [56]. Inflammatory reactions play important roles in tumor development, cancer promotion, and growth, as well as in invasion and metastasis [57]. In particular, urothelial cancer is known to be an immunogenic malignancy with tumor-infiltrating cytotoxic cells inducing BCa cell proliferation caused by the release of excessive amounts of cytokines [17]. This, together with the ease of access and measurement of inflammatory biomarkers in peripheral blood, make the latter an obvious candidate for prognostic biomarker research.

Nevertheless, it should be mentioned that studies evaluating biomarkers show an association between the marker and cancer outcomes rather than an actual predictive accuracy [15]. Since the use of a biomarker should add a benefit in clinical practice compared to an already existing standard model, internal and external validation is needed for all reported biomarkers. The incorporation of statistical tools such as decision curve analysis has not been performed in most studies, therefore limiting the use of these biomarkers in guiding therapeutic decisions [15].

Using meta-analysis methodology, we found an association between elevated NLR and decreased RFS. Urothelial cancer patients with preoperatively increased NLR exhibited increased rates of disease recurrence after radical surgery compared to patients with lower NLR. Except for one publication by Mari et al., six articles included in this meta-analysis reported a statistically significant negative correlation of elevated NLR with RFS [20,21,22,32,33,36,45]. These results support the integration of NLR for risk stratification tools and could help guide decision-making, especially regarding treatment intensification. Since patterns of NLR change are reported to vary significantly between responders and non-responders to systemic therapies, this ratio might also be investigated as a predictive tool to identify patients likely to respond to systemic therapy [58].

The NLR is calculated using peripheral blood measurements and reflects the balance between acute/chronic inflammation or adaptive immunity, as neutrophil counts rise in acute and chronic inflammatory reactions [59]. Besides urologic cancers, blood-based NLR was also shown to exhibit a prognostic value in gastrointestinal and gynecological cancers [60]. An isolated rise in neutrophils is also observed in bacterial and fungal infections, strokes and myocardial infarctions, atherosclerosis, and tissue damage that activates a systemic inflammatory response [61,62,63,64,65]. Although NLR is relevant for many different clinical scenarios, the cut-off value for its upper normal limit is still under debate. Beyond oncology, Song et al. reported an association of elevated NLR with overall mortality in the United States general population between 1999 and 2014 [59]. They also showed increased NLR values in patients that died due to heart disease, chronic lower respiratory disease, pneumonia, and kidney disease. Since NLR elevation can be caused by various maladies and circumstances, a very precise patient selection should be conducted in studies with survival outcomes as primary endpoints. Karakonstantis et al. reported several confounders in NLR values such as age, steroid intake, endogenous sexual hormones, and hematological disorders [66]. In a study by Fest et al., a significant difference in NLR values between female and male subjects was shown [67]. Hence, the above-mentioned factors might contribute to a bias in the results of conducted studies. A patient-selection approach for NLR utilization as a prognostic factor of recurrence after RC could be used to exclude patients with immunological or hematological diseases that could alter the NLR (i.e., leukemia, viral infections and chronic inflammatory diseases, malignant lymphoma, autoimmune diseases) as well as other malignancies [20,45]. Besides that, blood collection in patients receiving neoadjuvant chemotherapy should be carried out before the initiation of chemotherapy, since this could also be an influencing factor [22].

Our study is limited by several factors. NLR cut-off values differed between articles (ranging from 2.6 to 3.0), which makes the data less comparable. A consensus for standardized cut-off values has not been found yet. Additionally, every eligible article for this meta-analysis was conducted as a retrospective single- or multicenter study, thereby having an inferior level of evidence compared to prospective studies. The number of eligible articles for our meta-analysis was low, as only seven studies met our search criteria, and a high heterogeneity between these articles was found. Articles not written in English were excluded during the screening process, which could also lead to bias. In summary, prospectively conducted trials with larger sample sizes are needed to validate the most promising blood-based biomarker candidates that could be used to optimize risk stratification regarding treatment selection for patients with muscle-invasive BCa.

## 5. Conclusions

This study, focusing on disease recurrence, pooled available data on preoperative blood-based biomarkers as prognostic factors for survival outcomes in patients treated with RC for BCa. Several blood-based biomarkers such as inflammatory markers, factors of angiogenesis, circulating tumor cells and DNA, nutritional factors, and serum carbohydrate antigen seem to be promising tools for the prediction of recurrence after RC. Using a meta-analysis, we found a RFS benefit in patients with preoperatively measured NLR values below the upper limit of normal. However, to use these biomarkers as a part of a risk stratification tool in clinical decision-making, further investigation based on well-designed prospective clinical trials with larger sample sizes and strict patient inclusion criteria to reduce confounders are needed.

## Figures and Tables

**Table 1:** Eligible studies for the systematic review and their study characteristics.

Author	Year	Blood-Based Biomarker	Sample Size	Study Design	Survival Outcomes
Chang et al. [27]	2004	CA 125	n = 287	retrospective single-center	RFS, OS
Kouba et al. [28]	2009	CA 125	n = 92	prospective single-center	recurrence
Hazzaa et al. [29]	2010	clusterin	n = 26	prospective single-center	RFS, OS
Rink et al. [30]	2012	HER2 expression of circulating tumor cells	n = 100	prospective single-center	OS, CSS, RFS
Ahmadi et al. [31]	2014	CA 19-9, CA 125, CEA	n = 186	prospective single-center	OS, RFS
Hermanns et al. [32]	2014	NLR	n = 424	retrospective single-center	OS, CSS, RFS
Viers et al. [33]	2014	NLR	n = 899	retrospective single-center	OS, CSS, RFS
Djaladat et al. [34]	2014	albumin level, ASA score	n = 1471	retrospective single-center	RFS, OS
Morizawa et al. [22]	2016	NLR	n = 110	retrospective single-center	OS, CSS, RFS
Bhindi et al. [20]	2016	hemoglobin, individual cell counts, NLR, MLR, LMR, PLR	n = 418	retrospective single-center	OS, CSS, RFS
Liu et al. [35]	2016	albumin/globulin ratio	n = 296	retrospective multicenter	RFS, CSS
Tan et al. [36]	2017	NLR	n = 84	retrospective single-center	OS, CSS, RFS
D’Andrea et al. [21]	2017	LMR, NLR	n = 4198	retrospective multicenter	OS, CSS, RFS
Christensen et al. [37]	2019	circulating tumor DNA	n = 68	prospective single-center	RFS, OS
Yuk et al. [38]	2019	De Ritis ratio	n = 771	retrospective single-center	OS, CSS, RFS
Ninomiya et al. [39]	2020	NLR, MLR, LPR, De Ritis ratio, PNI	n = 107	retrospective single-center	OS, RFS
Fallah et al. [40]	2020	Myeloid-derived suppressor cells	n = 109	retrospective single-center	OS, RFS
Su et al. [41]	2020	lactate dehydrogenase	n = 263	retrospective single-center	OS, CSS, DFS
Dohn et al. [42]	2021	uPA	n = 107	prospective single-center	OS, CSS, RFS
Schuettfort et al. [43]	2021	albumin-globulin ratio	n = 4335	retrospective multicenter	OS, CSS, RFS
Li et al. [44]	2021	albumin-to-alkaline phosphatase ratio	n = 199	retrospective single-center	OS, CSS, RFS
Mari et al. [45]	2021	inflammatory biomarkers	n = 255	retrospective single-center	recurrence, CSM, OM
Schuettfort et al. [46]	2021	uPA, SuPAR, PAI-one	n = 1036	retrospective multicenter	RFS, CSS
Schuettfort et al. [47]	2022	panel of SIR markers	n = 4199	retrospective multicenter	CSS, RFS
Grossmann et al. [48]	2022	systemic immune-inflammation index	n = 4335	retrospective multicenter	OS, CSS, RFS
Laukhtina et al. [49]	2022	endoglin	n = 1036	retrospective multicenter	OS, CSS, RFS
Sari Motlagh et al. [50]	2022	IGF-I, IGFBP-2, IGFBP-3	n = 1036	retrospective multicenter	OS, CSS, RFS
Mori et al. [51]	2022	VCAM-1	n = 1036	retrospective multicenter	OS, CSS, RFS
Lei et al. [52]	2022	NLR, PLR, LMR	n = 186	retrospective single-center	RFS
Schuettfort et al. [53]	2022	Interleukin-6 and its soluble receptor	n = 1036	retrospective multicenter	OS, CSS, RFS
Urabe et al. [54]	2022	Serum microRNA	n = 81	retrospective single-center	OS, PFS
Katayama et al. [55]	2022	hepatocyte growth factor	n = 565	retrospective multicenter	OS, CSS, RFS
Mori et al. [51]	2022	VEGF plasma levels	n = 1036	retrospective multicenter	OS, CSS, RFS

uPA = urokinase-type plasminogen activator, CA = carbohydrate antigen, CEA = carcinoembryonic antigen, HER = human epidermal growth factor receptor, SIR = systemic inflammatory response, LMR = lymphocyte-to-monocyte ratio, NLR = neutrophil-to-lymphocyte ratio, IGF = insulin-like growth factor, IGFBP = insulin-like growth factor-I binding protein, suPAR = urokinase-type plasminogen activator soluble receptor, PAI = urokinase-type plasminogen inhibitor, VCAM = vascular cell adhesion molecule, MLR = monocyte-to-lymphocyte ratio, LPR = lymphocyte-to-platelet ratio, PNI = prognostic nutritional index, PLR = platelet-to-lymphocyte ratio, ASA = American Society of Anesthesiologists, OS = overall survival, CSS = cancer-specific survival, RFS = recurrence-free survival, CSM = cancer-specific mortality, OM = overall mortality, VEGF = vascular endothelial growth factor, PFS = progression-free survival, DFS = disease-free survival.

**Table 2:** Eligible studies for the meta-analysis and their study characteristics.

Author	Year	Sample Size	Age, Years, Median (Range)	Pathological Stage	Median Follow-Up (Range)	HR, Multivariate Analysis (CI)	NLR Cut-Off	*p*-Value
Viers et al. [33]	2014	899	69 (62–76)	T ≤ 1–T4, Nx, N0–N3	10.9 years (8.3–13.9)	Recurrence: HR 1.04 (1.01–1.08)	NLR > 2.7	*p* = 0.02
Hermanns et al. [32]	2014	424	70.1 (60.6–76.3)	T0–T4, Ta, Tis, Nx, N0, N+	58.4 months (21.3–94.5)	Recurrence: HR 1.49 (1.12–2.00)	NLR ≥ 3	*p* = 0.007
Bhindi et al. [20]	2016	418	70 (61–76)	T0– T4, Ta, Tis, N0, N+, Nx	40 months (14–72)	RFS: HR 1.52 (1.17–1.98)	NLR < 2.9	*p* = 0.002
Morizawa et al. [22]	2016	110	72 (65–76)	T0–T4, N+	37.5 months (11–65)	RFS: HR 2.6 (1.1–6.0)	NLR < 2.6	*p* = 0.02
D’Andrea et al. [21]	2017	4198	67 (60–73)	T0–T4, Ta, Tis; N+	not reported	RFS: HR 1.2 (1.1–1.3)	NLR < 2.7	*p* < 0.001
Tan et al. [36]	2017	84	67 (37–82)	T1–T4, N+	30.1 months (3.2–161.7)	Recurrence: HR 6.999 (1.712–28.606)	NLR ≥ 2.7	*p* = 0.007
Mari et al. [45]	2021	255	79 (75–83)	T1–T4, Nx, N0–N3	Not reported	Recurrence: HR 1.13 (0.96–1.32)	NLR > 3	*p* = 0.14

HR = hazard ratio, CI = confidence interval, NLR = neutrophil-to-lymphocyte ratio, RFS = recurrence-free survival.

## Data Availability

Data used in this systematic review and meta-analysis is cited in the references. Used electronic databases include PubMed (https://pubmed.ncbi.nlm.nih.gov) and Scopus (https://www.scopus.com).

## Supplementary Materials

The following supporting information can be downloaded at: https://www.mdpi.com/article/10.3390/ijms24065846/s1.
