# Supplementary material for: Blood-Based Biomarkers as Prognostic Factors of Recurrent Disease after Radical Cystectomy: A Systematic Review and Meta-Analysis
Source: Int J Mol Sci. 2023 Mar 19;24(6):5846. doi: 10.3390/ijms24065846 (PMC10056816; doi:10.3390/ijms24065846)
Supplement: Supplementary file 1 [file ijms-24-05846-s001.zip › Table S1_ROBINS-I.pdf]

Table 1: Risk Of Bias In Non-randomized Studies of Interventions (ROBINS-I)[1]:

Author	Confounding	Participant selection	Classification of interventions	Deviation from intended intervention	Missing data	Measurement of outcomes	Selection of the reported result	Overall
Mari et al.[2], 2021	Moderate	Low	Low	Low	Low	Moderate	Low	Low
D'Andrea et al [3]., 2017	Moderate	Low	Low	Moderate	Moderate	Moderate	Low	Moderate
Morizawa et al.[4], 2016	Moderate	Serious	Moderate	Moderate	Low	Moderate	Low	Moderate
Viers et al.[5], 2014	Moderate	Moderate	Moderate	Moderate	Moderate	Moderate	Moderate	Moderate
Tan et al.[6], 2017	Moderate	Moderate	Low	Low	Moderate	Low	Moderate	Moderate
Hermanns et al.[7], 2014	Moderate	Moderate	Moderate	Moderate	Moderate	Low	Low	Moderate
Bhindi et al.[8], 2016	Moderate	moderate	Moderate	Moderate	Moderate	Moderate	Low	Moderate

References

1. Sterne JA, Hernán MA, Reeves BC, et al. ROBINS-I: a tool for assessing risk of bias in non-randomised studies of interventions. *BMJ*. 2016;355:i4919. doi:10.1136/bmj.i4919

2. Mari A, Muto G, Di Maida F, et al. Oncological impact of inflammatory biomarkers in elderly patients treated with radical cystectomy for urothelial bladder cancer. *Arab J Urol*. 2021;19(1):2-8. doi:10.1080/2090598X.2020.1814974

3. D'Andrea D, Moschini M, Gust KM, et al. Lymphocyte-to-monocyte ratio and neutrophil-to-lymphocyte ratio as biomarkers for predicting lymph node metastasis and survival in patients treated with radical cystectomy. *J Surg Oncol*. 2017;115(4):455-461. doi:10.1002/jso.24521

4. Morizawa Y, Miyake M, Shimada K, et al. Neutrophil-to-lymphocyte ratio as a detection marker of tumor recurrence in patients with muscle-invasive bladder cancer after radical cystectomy. *Urol Oncol Semin Orig Investig*. 2016;34(6):257.e11-257.e17. doi:10.1016/J.UROLONC.2016.02.012

5. Viers BR, Boorjian SA, Frank I, et al. Pretreatment neutrophil-to-lymphocyte ratio is associated with advanced pathologic tumor stage and increased cancer-specific mortality among patients with urothelial carcinoma of the bladder undergoing radical cystectomy. *Eur Urol*. 2014;66(6):1157-1164. doi:10.1016/J.EURURO.2014.02.042

6. Tan YG, Eu E, Lau Kam On W, Huang HH. Pretreatment neutrophil-to-lymphocyte ratio predicts worse survival outcomes and advanced tumor staging in patients undergoing radical cystectomy for bladder cancer. *Asian J Urol*. 2017;4(4):239-246. doi:10.1016/j.ajur.2017.01.004

7. Hermanns T, Bhindi B, Wei Y, et al. Pre-treatment neutrophil-to-lymphocyte ratio as predictor of adverse outcomes in patients undergoing radical cystectomy for urothelial carcinoma of the bladder. *Br J Cancer*. 2014;111(3):444-451. doi:10.1038/bjc.2014.305

8. Bhindi B, Hermanns T, Wei Y, et al. Identification of the best complete blood count-based predictors for bladder cancer outcomes in patients undergoing radical cystectomy. *Br J Cancer*. 2016;114(2):207-212. doi:10.1038/bjc.2015.432
