# Supplementary material for: Blood-Based Biomarkers as Prognostic Factors of Recurrent Disease after Radical Cystectomy: A Systematic Review and Meta-Analysis
Source: Int J Mol Sci. 2023 Mar 19;24(6):5846. doi: 10.3390/ijms24065846 (PMC10056816; doi:10.3390/ijms24065846)
Supplement: Supplementary file 1 [file ijms-24-05846-s001.zip › Table S2_Study characteristics_MA.pdf]

Table 2: Study characteristics, Meta-analysis

Author	Year	Sample Size	Gender (%female)	Age, years, median (range)	Median Follow up (range)	HR, multivariate analysis (CI)	NLR cut-off	p-value	Timepoint NLR measurement	Neoadjvant chemotherapy, n (%)	previous radiotherapy, n (%)
Mari et al.[1]	2021	255	16,90%	79 (75–83)	not reported	Recurrence: HR 1.13 (0.96–1.32)	NLR > 3	p = 0.14	preoperative	none (patients excluded, n = 19)	4 (1.6)
D'Andrea et al.[2]	2017	4198	20%	67 (60-73)	not reported	RFS: HR 1.2 (1.1-1.3)	NLR < 2.7	p < 0.001	preoperative	none (patients excldued)	not reported
Morizawa et al.[3]	2016	110	22%	72 (65-76)	37.5 months (11-65)	RFS: HR 2.6 (1.1-6.0)	NLR < 2.6	p = 0.02	preoperative	37 (34)*	not reported
Viers et al.[4]	2014	899	19,60%	69 (62-76)	10.9 years (8.3 - 13.9)	Recurrence: 1.04 (1.01–1.08)	NLR > 2.7	p = 0.02	preoperative	none (patients excluded, n = 53)	none (Patients excluded, n = 13)
Tan et al.[5]	2017	84	25%	67 (37-82	30.1 months (3.2 - 161.7)	Recurrence: 6.999 (1.712 - 28.606)	NLR ≥ 2.7	p = 0.007	preoperative	none (patients excluded, n = 16)	not reported
Hermanns et al.[6]	2014	424	23,40%	70.1 (60.6-76.3)	58.4 months (21.3-94.5)	Recurrence: HR 1.49 (1.12–2.00)	NLR ≥ 3	p = 0.007	preoperative	29 (6.8)	salvage RC patients following failed chemoradiation were excluded (n = 20)
Bhindi et al.[7]	2016	418	23%	70 (61-76)	40 months (14-72)	RFS: HR 1.52 (1.17 - 1.98)	NLR < 2.9	p = 0.002	preoperative	28 (7)	salvage RC patients following failed chemoradiation were excluded (n = 20)

*laboratory parameters were defined the date of initiation of neoadjuvant chemotherapy

NLR = neutrophil-to-lymphocyte ratio; RFS = recurrence free survival; HR = hazard ratio; RC = radical cystectomy; CI = confidence interval

References

1. Mari A, Muto G, Di Maida F, et al. Oncological impact of inflammatory biomarkers in elderly patients treated with radical cystectomy for urothelial bladder cancer. Arab J Urol. 2021;19(1):2-8. doi:10.1080/2090598X.2020.1814974

2. D'Andrea D, Moschini M, Gust KM, et al. Lymphocyte-to-monocyte ratio and neutrophil-to-lymphocyte ratio as biomarkers for predicting lymph node metastasis and survival in patients treated with radical cystectomy. J Surg Oncol. 2017;115(4):455-461. doi:10.1002/jso.24521

3. Morizawa Y, Miyake M, Shimada K, et al. Neutrophil-to-lymphocyte ratio as a detection marker of tumor recurrence in patients with muscle-invasive bladder cancer after radical cystectomy. Urol Oncol Semin Orig Investig. 2016;34(6):257.e11-257.e17. doi:10.1016/J.UROLONC.2016.02.012

4. Viers BR, Boorjian SA, Frank I, et al. Pretreatment neutrophil-to-lymphocyte ratio is associated with advanced pathologic tumor stage and increased cancer-specific mortality among patients with urothelial carcinoma of the bladder undergoing radical cystectomy. Eur Urol. 2014;66(6):1157-1164. doi:10.1016/J.EURURO.2014.02.042

5. Tan YG, Eu E, Lau Kam On W, Huang HH. Pretreatment neutrophil-to-lymphocyte ratio predicts worse survival outcomes and advanced tumor staging in patients undergoing radical cystectomy for bladder cancer. Asian J Urol. 2017;4(4):239-246. doi:10.1016/j.ajur.2017.01.004

6. Hermanns T, Bhindi B, Wei Y, et al. Pre-treatment neutrophil-to-lymphocyte ratio as predictor of adverse outcomes in patients undergoing radical cystectomy for urothelial carcinoma of the bladder. Br J Cancer. 2014;111(3):444-451. doi:10.1038/bjc.2014.305

7. Bhindi B, Hermanns T, Wei Y, et al. Identification of the best complete blood count-based predictors for bladder cancer outcomes in patients undergoing radical cystectomy. Br J Cancer. 2016;114(2):207-212. doi:10.1038/bjc.2015.432
